# A quality dietary supplement: before you start and after it’s marketed—a conference report

**DOI:** 10.1007/s00394-014-0827-4

**Published:** 2015-01-13

**Authors:** Mark A. LeDoux, Kristy R. Appelhans, Lesley A. Braun, Darren Dziedziczak, Sam Jennings, Laura Liu, Henry Osiecki, Edward Wyszumiala, James C. Griffiths

**Affiliations:** 1 Natural Alternatives International, Inc., San Marcos, CA USA; 2 Herbalife International of America, Inc., Torrance, CA USA; 3 Blackmores Institute, Sydney, Australia; 4 Blackmores, Ltd., Warriewood, Sydney, Australia; 5 Berry Ottaway and Associates, Ltd., Hereford, UK; 6 NSF-International, Shanghai, China; 7 Bioconcepts, Warwick, Australia; 8 GMP Auditing Partners, Akron, OH USA; 9 Council for Responsible Nutrition, Washington, DC USA

## Abstract

Consumers worldwide are turning to dietary supplements as one part of their personal goal to lead healthier and more active lives. In truth, the quality of life now supersedes the length of life as no one would trade living to one hundred (the last forty with compromised physical abilities and decreased mental acuity) for 80 years of travel, time with family, and intellectual pursuits. If there is the possibility of preventing a disease or debilitating condition through efficient lifestyle changes (additions, subtractions, modifications) and to also avoid the costly and escalating medical and pharmaceutical treatments that accompany having the disease/condition, then a sensible individual would focus on their overall health and wellness…proactively, instead of reactively. However, an important caveat is that over-regulation or inappropriate application of current regulations can increase the price of dietary supplements and nutritional products and thus cause underutilization of the potentially beneficial physiological attributes of these products. Conversely, strict adherence to regulatory guidelines could result in safer dietary supplements and fewer adverse reactions requiring medical attention. If new regulations or stricter interpretation/application of existing regulations result in certain dietary supplements being taken off the market, will continued demand create a completely unregulated, underground economy that will create unforeseen problems? More research should be supported by government agencies to determine the effectiveness of dietary supplements, nutritional products and complementary medicine in reducing personal and societal medical costs and further contribution to the overall health of the population.

Since the completion of the good manufacturing practices (GMPs) regulations in the USA in 2007, the dietary supplements industry has continued to experience solid growth, thanks in part to a regulatory structure that allows new products a quick time-to-market. In turn, consumers have benefitted from the wide range and availability of dietary supplements that help support a healthy lifestyle. However, with this opportunity and ease of the open market comes a great deal of responsibility to ensure that dietary supplements are safe and meet the quality standards that consumers, and regulators, expect. The industry that is engaged in producing dietary supplements and ancillary nutritional products, (sometimes termed differently in other regions of the world) now more than ever need to strengthen their quality control systems to prevent adulterated goods from entering commerce. To achieve that end, industry also needs to work collaboratively with national and regional regulators. These steps would serve to create early warning systems to identify raw materials moving through the supply chain that are at risk for contamination, adulteration or poor quality. Further, when excessive demand, rising prices and limited availability surround key ingredients or materials of sudden interest, then substandard quality though accident or intentionality become more common and harder to detect. Responsible manufacturers at both the initial “dietary ingredient” and final dosage form “dietary supplement” experience a number of anomalous findings in expected parameters for quality through the rigorous application of in-house hazard analysis and critical control point (HACCP) and GMP systems of incoming raw material qualification and compliance.

Post-manufacturing issues related to quality include aspects of stability and shelf life over the course of the expected consumer availability of each formula and lot number. Post-market adverse event data are a critical component of the product safety assessment process and allows for ethical and responsive stewardship by the manufacturer for each and every item placed on the market. It is important to understand how this information may be utilized to establish best practices for post-market data collection, documentation and communication.

## Good manufacturing practices (GMPs)

 Dietary supplements as a product category was officially defined in the US with the passage of the Dietary Supplements Health and Education Act (DSHEA) in 1994 and since then, a series of regulatory requirements and provisions have been rolled out and enforced by the Food and Drug Administration (FDA) under this regulatory framework. There are GMP requirements specific to the safety and quality of a product related to the manufacturing, packaging and labeling for all foods, dietary supplements and pharmaceutical products. The process control approaches to ensure safety and quality assurance, such as quality verification and validation of the product and process are raised or highly recommended by GMP regulation or international quality management schemes such as the International Organization for Standardization’s Quality Management System (ISO9001) [10], Safe Quality Food (SQF) [17] Certification and HACCP [6]. Unfortunately, FDA-issued warning letters in recent years show that many of the smaller firms are still struggling to comply with these regulations.

 The US model for GMP regulations is unique, and although specific for the manufacturing of dietary supplements, it is in reality a hybrid of food and pharmaceutical GMPs. The dietary supplement regulations define the manufacturing and quality requirements for all supplements sold and distributed in the US market. Identity testing on all incoming batches of raw materials is required, thereby assuring that companies are verifying that the raw materials purchased are truly the intended materials. According to FDA warning letters and other forms of communication, many firms are still not spending enough time developing appropriate test methods to validate the identity of incoming ingredients. To satisfy this GMP requirement, many companies use in-house verification technology, such as Fourier transform infrared (FTIR) spectroscopy and near-infrared (NIR) spectrometers. These technologies are commonly used, as they are relatively inexpensive and quick, hence their appeal; however, they may not be appropriate for identifying all incoming materials, such as blends or botanicals. The more complex the ingredient, the more likely the complexity of the fingerprint is anticipated and observed. The most important aspect is establishing a reference standard of what a sample of good, high-quality material should look like; i.e., companies need these reference standards with which to compare incoming ingredients. An FTIR and NIR spectrometer will produce a scan for virtually any ingredient, but without an established standard to compare an ingredient against, it is hard to tell whether the incoming ingredient meets the manufacturer’s specification. To properly develop these reference standards, companies should have several samples (six or more, preferably) that they have qualified using other techniques, to develop a library of good ingredient data sets. NIR instruments in particular are sensitive enough to pick up very subtle differences in various ingredient lots, which may be particularly critical when dealing with botanicals. Using a baseline sample of the targeted material for subsequent identification verification is an incorrect method for establishing reference standards, which should be separately obtained, qualified and validated.

Another area the regulation has implemented is that companies can no longer just simply rely on the raw material suppliers’ certificate of analysis (CoA). Companies have to assume the responsibility for the accuracy of the CoA through testing to verify the accuracy of the data and qualify the data against the company’s own internal specification for each raw material. GMPs require that dietary supplement companies qualify their suppliers if they are going to rely on the data on the CoA. Unfortunately, dietary ingredients and their CoAs can pass through many hands, especially if brokers of raw materials are used, before making their way to a company’s facility for processing, providing plenty of opportunity for contamination and/or fraud to occur. Depending on the nuances of a company’s supply chain, it may not be enough to just compare a CoA against expected specifications. CoAs may not always be a reliable testament to the quality of the ingredient, and proper testing to verify the source may help eliminate this type of adulteration.

Once products are manufactured, consumers place a great deal of trust in the recognizable brand and expect product labels to be accurate. Testing finished products to verify the accuracy of their labels, as well as the absence of harmful levels of contaminants such as lead, is paramount to GMP compliance; however, testing complex finished products requires high-tech instrumentation, test methods and expertise that many companies do not have in house. Some finished products may have upwards of 20 different ingredients in a unique formulation. The more complex the formula, the more complex the testing and verification often involving trace amounts, i.e., microgram and milligram quantities—leading to testing difficulties in sample preparation. The challenge can sometimes lie in selecting the appropriate test methods and instruments. Companies that manufacture complex proprietary blends have an even greater challenge, as there are no compendial standards to compare their finished products against (as opposed to a product containing a singular ingredient). This is why many companies seek the expertise of outside laboratories to help review their formulas and develop test methods, or even perform the testing for them.

The GMP regulations have established the foundation for quality in the US market. The long-term effect of this regulation will only continue to promote growth and even higher-quality ingredients and products.

## Verification and validation

There is confusion with the terms “verification” and “validation,” and any distinction is not overly apparent to some manufacturers or auditors. This confusion makes it difficult to implement the process controls completely and effectively to ensure that the product meets the quality needs and keeps up with continued improvement. For example, verification in a HACCP [6] system is “The application of methods, procedures, tests and other evaluations, in addition to monitoring to determine compliance with the HACCP plan.” This definition illustrates some verification activities such as procedure or protocol review, inspection or examination, tests followed and fulfilled, and records review. In short, verification emphasizes that the procedures and control measures were followed appropriately, while validation seeks the evidence to prove these control measures actually worked and were effective. Verification could be part of the process of validation. When direct verification activities such as in-process and finished product inspection and testing cannot be adequately conducted to assure the quality, validation is needed.

Even though the regulations for dietary supplement GMPs in the US do not require that the formal validation protocol be adopted in the quality system, the systemic approach of verification and validation is recommended to be applied to the critical stages of the dietary supplement manufacturing process, since many of these manufacturers have limited resources and inadequate technology to do all of inspection and testing for both in-process and finished products. The control measures at these critical stages or points could be identified through a risk analysis approach using HACCP principles to quantify and rank the risk. The control measures applied to these points should be monitored or verified by trained and qualified person using well-functioned equipment and following applicable procedures. In the manufacturing chain for dietary supplement, supplier control and the examination or testing of raw materials per the specifications for production is normally considered to be one of the most critical points to control as any deviation would drastically impact product quality including the identity, strength, composition and any potential contaminants. The first step is to determine and establish the specifications for the raw materials based on scientific data and regulatory requirements, as well as the requirements to meet the intended use. Before the suppliers of these raw materials are approved, they must be carefully evaluated with the consideration of CoA compliance, the supplier’s quality systems and other non-quality concerns, such as supplier history and reputation in the qualification process [15]. During the manufacturing process for dietary supplements, critical production processes, such as heating, cooling, weighing and formulation, specific cleaning and sanitizing operations need to be validated in accordance to guidelines of the FDA and/or Health-Canada [8, 20].

For both verification and validation, the process needs to be planned and organized in advance by trained and qualified personnel. The activities should be carefully followed according to the written procedures and well documented.

## Supplier compliance

Suppliers, including contract manufactures, are a critical link in the dietary supplement and nutritional product supply chain. If a raw material supplier or contract manufacturer does not align with your “quality values,” then your product quality and ultimately brand reputation could be adversely impacted. Supplier compliance should be a key element in your quality management system and supports the concept of quality by design by ensuring quality is built into products.

Any supplier who has the potential to impact product quality should be managed in a structured way to minimize risk to product quality. The supplier compliance management process is made up of three main elements: (1) approving a potential supplier; (2) monitoring performance, followed by (3) periodic on-site reviews.

After identifying a potential manufacturer, the first step should be to assess and approve this supplier as being suitable to provide the required service. An assessment needs to be made based on either a GMP Questionnaire or an on-site GMP audit.

A quality GMP audit is defined as a systematic, independent and documented process to examine specific activities relating to the physical environment, systems and processes which impact the product. Specifically, the audit should verify the following objectives: (1) Appropriate processes and systems are in place; (2) processes and systems have been implemented successfully; and (3) processes and systems are being followed effectively. The outcome of the audit must provide sufficient evidence to give a clear indication that the supplier aligns with the expected quality objectives. An auditor must recognize that suppliers vary in size, scope and sophistication, and each site requires specific interpretation for their compliance to required quality standards. The three main areas of focus of an on-site audit are (1) facilities and processes, including physical areas of the operations which may directly or indirectly impact product quality; (2) quality systems, including compliance to those systems; and (3) product/service specific focus.

Typical audit techniques include a *vertical audit* which follows a process through a number of related systems or a *horizontal audit* which focuses on one system in a significant amount of detail. Generally, a combination of the two should be used in a quality audit. The main objectives of auditing are to determine whether the facility and suppliers processes have the capabilities required, while also ensuring that appropriate quality systems have been implemented and the supplier is compliant to these.

The auditing process should include a written report which documents the audit findings and provides some level of categorization of the non-conformance. Responses to the non-conformances should include both corrective actions and preventative actions. These responses must be reviewed and accepted before the audit can be closed. Based on this, a supplier will be either approved or rejected as a supplier.

Once a supplier has been approved, a program on monitoring quality performance with the supplier should be initiated. This should include agreed measures such as deviations, non-conformances, corrective and preventitive actions (CAPAs). This should be reported and discussed with the supplier periodically. Lastly, a plan for periodic re-audit or review of the supplier should be in place. This should be based on the risk profile of the supplier and include a review of actual batches manufactured and supplied to you.

Implementing a supplier compliance management system provides many benefits in enhancing product quality, including risk mitigation, surety of supply, a focus on continuous improvements, rapid issue resolution and strong confidence in the products supplied. Working closely and collaboratively with suppliers in this way helps support a close ongoing relationship with clear expectations. These elements in turn ensure that product quality is built into every batch of product.

### Stability and shelf life

Owing to their similarities in presentation, there is a tendency for government authorities to consider that all requirements for medicines are equally applicable to supplements, and this includes the issue of stability testing. For medicines, stability is essentially a safety concern, as stability testing ensures the safety and consistency of delivery of the drug over time. Conversely, for supplements, stability is essentially a quality concern. Stability testing enables the manufacturer to predict an appropriate shelf life for the product, thus ensuring that the consumers’ expectations of quality are met throughout the shelf life. The essential requirements of supplement stability are to ensure that no untoward organoleptic changes take place during the proposed life of the product and also to ensure that the product meets the quantitative requirements for the claimed active ingredients throughout its proposed shelf life.

In contrast to medicines, which are generally based on only one or two active ingredients, many supplements contain multiple active ingredients, a number of which may be inherently unstable. For example, in a multivitamin and mineral product, some of the vitamins will be more stable than others, and the rate of loss of activity under specified conditions will vary greatly from vitamin to vitamin, as vitamins do not all follow the same rules of thermodynamics. In addition, the form in which the vitamins are present can have an effect on their stability. There are over 40 forms of vitamins authorized for use in food supplements within the European Union, each of which has a different stability profile [5].

Supplement stability is affected by environmental factors, such as temperature, oxygen, moisture and ultraviolet light. As a generalization, approximately every 10 °C rise in temperature leads to a doubling of the rate of chemical reaction; thus, temperature can be an important factor in supplement stability. The presence of oxygen may also have a major impact on the stability of certain vitamins. Significant changes have been observed in the vitamin content of supplements stored in clear glass bottles when compared to the same formulation stored in near-identical amber glass bottles, as certain vitamins are particularly sensitive to the ultraviolet component of light.

The stability of supplements is also affected by other factors that are specific to the product. For example, the pH (high or low) can be a particularly critical factor in liquid products, while the presence of oxidizing and reducing agents can affect a number of active ingredients, especially vitamins (e.g., thiamin, folic acid). The water activity of a product can play an important role, for example, in microbiological stability. Metallic ions, such as copper or ferrous ions, can also play an important role in the instability of certain vitamins. There are also a number of vitamin–vitamin interactions or interactions between vitamins and other ingredients that can lead to loss of activity.

The environmental factors can generally be mitigated by the selection of appropriate packaging, but it is essential that a critical evaluation of a prospective formulation be undertaken before proceeding with manufacture, to ensure that all other potential factors that may affect its stability have been minimized. To achieve a commercially realistic shelf life, it is often necessary to add an additional quantity of some active ingredients during manufacture, to compensate for loss during storage. This additional amount is known as an “overage,” and it is normally expressed as a percentage of the declared level. For example, if the input level of an ingredient is 45 mg, and the declared level is 30 mg, the overage would be 50 %. The amount of overage required varies according to the known stability of the ingredient in the product matrix, but the total quantity of input of the ingredient must be within known safety limits or any set maximum levels. The concept of overages is well recognized in the supplement and fortified food sectors [2].

When undertaking a stability evaluation, many tests will be product specific, but will generally fall within one of four categories: (1) sensory/organoleptic evaluation (e.g., color, odor, taste), (2) chemical analysis (e.g., assays for levels of active components), (3) physical analysis (e.g., assessing the hardness of tablets), and (4) microbiological examination (based on assessments of microbiological risks). The tests selected for any given ingredient or product will depend on factors such as the nature and specifications of the product and the ingredient(s), the proposed label claims, the packaging and anticipated storage conditions and the shelf life required for commercial viability.

Two main types of stability studies are generally used for predicting shelf life for supplements: “Real time” studies run for the anticipated shelf life of the product and simulate the anticipated ambient storage conditions, whereas “accelerated studies” use elevated temperatures to speed up the rates of chemical reactions, over a shorter period of time [9]. Although the “real time” studies would be expected to provide more accurate shelf life data, especially “in use” studies (where one daily dose is removed from the package each day of the study, to simulate actual usage of the product over time), such studies are not always commercially viable before product launch. Therefore, most companies will have to utilize accelerated studies to obtain the initial shelf life indication. All shelf life tests should be carried out in the packaging that is to be used commercially. In cases where more than one packaging size is expected, such as 30- and 60-unit containers, the tests should be carried out on both containers. However, the suitability of the proposed packaging under commercial packaging conditions should be assessed prior to the stability tests being undertaken (e.g., checking to ensure seal integrity).

The selected shelf life should be supported and justified by relevant data, as this ensures the accuracy of the shelf life expiry date and also shows “due diligence”; i.e., the manufacturer can prove there is sound reasoning behind the determination of the expiry data. When determining the shelf life of a supplement, the manufacturer can take into account data from a range of sources. For example, they may utilize data from an appropriate stability study on the specific product; an extrapolation of data from stability studies on similar products; bibliographical references from scientific literature relating to the stability of the ingredients; or combinations of these data sources. Flexibility is needed when determining the shelf life for multi-active products, so all available and relevant data can be used to indicate the stability of such products [9].

### Adverse event reporting

Global adverse event reporting (AER) systems allow for the collection of relevant data that would allow a dietary supplement company to monitor product safety and quality worldwide and make continuous improvements as necessary. AER-related quality issues can identify product QA/QC failures that necessitate changes in formulations or in rare cases lead to product recalls. Integrating post-market AER monitoring with product quality complaints ensures the most comprehensive investigative practices and allows sensitive signal detection. An increasing number of governments have mandatory collection and reporting requirements for AEs, but signal detection methodologies are poorly defined and are designed for drugs and not nutritional products. New regulations for reporting are being developed and enforced worldwide, and regulatory authorities are now more frequently asking for adverse event information upon registration or re-registration of products. Systematic review of AE data provides manufacturers as well as regulatory authorities the necessary information to determine whether there are causal relationships with the use of specific products or product ingredients and adverse health outcomes.

The confinements which are inherent to pre-market product analyses (including clinical trials) and/or the lack of extensive pre-market clinical testing required for dietary supplements leaves post-market adverse event monitoring as the only practical way to evaluate the quality, safety and efficacy of dietary supplements after they are introduced into the marketplace.

Primary uses for post-market AER data include (1) compliance with global regulations for post-market surveillance/“pharmacovigilance”; (2) support a company’s ongoing product quality/safety evaluations; and (3) support external communications regarding the safety of a company’s product portfolio. Systematic data collection and review processes, standardized nomenclature, and analytical technology are crucial components for producing meaningful AER data trends and conducting signal detection.

Future considerations include developing standardized/globally accepted causality assessment criteria which is specific to complex substances (i.e., multi-ingredient dietary supplements) to reduce generalization which contributes to inaccurate conclusions regarding the safety of supplements and/or their ingredients. Adverse reactions can happen with exposure to any substance; however, there are extremely few serious adverse reactions to dietary supplements, nutritional products and over-the-counter complementary medicines. This is likely to be due to the inherent safety of the ingredients, high-quality control standards and stringent regulations.

### Post-market surveillance

One of the most frequent criticisms of dietary supplement regulations and basis for scrutiny around the safety of such products is that there is often no pre-market clinical testing required which would have required that preliminary safety assessments be conducted. However, regulatory authorities often require that a dietary supplement product be registered or licensed with documentation substantiating the safety, scientific rationale for claims, and quality specifications for the finished good and/or individual ingredients.

Additionally, it is important to acknowledge certain deficiencies which are common to any pre-market analysis (for drugs or dietary supplements) including sample populations which are not an accurate representation of the population at large and therefore may not account for relevant factors which can affect the expectedness of certain events or outcomes such as medication use, preexisting medical conditions, metabolic differentiation and/or lifestyle considerations (e.g., diet, tobacco or alcohol use). In fact, the frequency at which drug products are recalled post-market for safety reasons despite extensive pre-market research and clinical testing exemplifies some of the limitations on pre-market safety assessments. Several studies have been conducted to demonstrate this point, and in 2001, a retrospective review of 150 drug withdrawals revealed the most frequent safety reasons for removal from the market of which included several serious concerns such as hepatotoxicity (27.9 %), cardiovascular toxicity (17.4 %), nephrotoxicity (5.6 %), neurotoxicity (6.3 %) and carcinogenicity (6.3 %) [11, 13].

Therefore, the confinements which are inherent to pre-market product analyses and/or the lack of extensive pre-market clinical testing required for dietary supplements leaves post-market adverse event monitoring as the only practical way to evaluate the quality, safety and efficacy of dietary supplements after they are introduced into the marketplace.

AE data may be used to communicate with regulatory authorities. Often such communications are related to pharmacovigilance/post-market surveillance compliance obligations and may include expedited reporting, periodic safety update reports (PSURs), or spontaneous inquiries or inspections concerning AERs [7, 18, 19]. As mentioned above, product registration requirements may also require an AER statement or other related safety disclosures accompany the marketing application.

Post-market safety data also helps to provide accurate and appropriate responses to consumer inquiries. Like AEs, consumer inquiries may help inform product safety reviews by providing additional insight regarding the product’s target demographic such as the prevalence of certain medical conditions or use of medications, age distribution, lifestyle trends, relevant cultural practices, and gender.

Perhaps more frequently than not, dietary supplements are under tremendous scrutiny in regard to safety [4]. In many of these instances, the allegations are unsubstantiated. However, a robust post-market safety surveillance program will help a company respond to such matters with objective information which may include disclosure of exposure and incidence data that clearly negate any alleged causal associations.

Internally, post-market data can also be used to support a company’s ongoing product quality/safety evaluations. This information is essential for conducting informed risk assessments which may be applicable to product recall or withdrawal decisions, labeling revisions which may include warnings or precautionary statements, and/or product reformulation strategies.

Future considerations in post-market safety surveillance for dietary supplements include developing standardized/globally accepted causality assessment criteria which is specific to complex substances (i.e., multi-ingredient dietary supplements) to reduce generalization which contributes to inaccurate conclusions regarding the safety of supplements and/or their ingredients.

## Conclusion

The word “quality” has three meanings in my Merriam-Webster dictionary [14]; all three are germane to the discussion in this article. (1) Quality is a noun describing “how good or bad something is.” (2) Quality is a noun describing “a characteristic or feature that someone or something has; something that can be noticed as a part of a person or thing.” (3) Quality is a noun describing “a high level of value or excellence.” In summary, the “quality” of a dietary supplement of nutritional product can either be “good” or “bad,” objectively measured via analytical parameters. Dietary supplements and nutritional products have an inherent “quality,” i.e., a feature that is descriptive of the product as desired, purchased and used. Finally, a “quality” dietary supplement and nutritional product is one with a high level of value or excellence…it is the product the consumer wants.
